# ADAM17 Mediates MMP9 Expression in Lung Epithelial Cells

**DOI:** 10.1371/journal.pone.0051701

**Published:** 2013-01-14

**Authors:** Ya-qing Li, Jian-ping Yan, Wu-lin Xu, Hong Wang, Ying-jie Xia, Hui-jun Wang, Yue-yan Zhu, Xiao-jun Huang

**Affiliations:** 1 Department of Respiratory Medicine, Zhejiang Provincial People's Hospital, Hangzhou, China; 2 Zhejiang Provincial Gastroenterology Key Laboratory, Zhejiang Provincial People's Hospital, Hangzhou, China; University of Western Ontario, Canada

## Abstract

The purposes were to study the role of lipopolysaccharide (LPS)-induced tumor necrosis factor (TNF)-α/nuclear factor-κB (NF-κB) signaling in matrix metalloproteinase 9 (MMP9) expression in A549 cells and to investigate the effects of lentivirus-mediated RNAi targeting of the disintegrin and metalloproteinase 17 (*ADAM17*) gene on LPS-induced MMP9 expression. MMP9 expression induced by LPS in A549 cells was significantly increased in a dose- and time-dependent manner (*p*<0.05). Pyrrolidine dithiocarbamate (PDTC) and a TNFR1 blocking peptide (TNFR1BP) significantly inhibited LPS-induced MMP9 expression in A549 cells (*p*<0.05). TNFR1BP significantly inhibited LPS-induced TNF-α production (*p*<0.05). Both PDTC and TNFR1BP significantly inhibited the phosphorylation of IκBα and expression of phosphorylation p65 protein in response to LPS (*p*<0.05), and the level of IκBα in the cytoplasm was significantly increased (*p*<0.05). Lentivirus mediated RNA interference (RNAi) significantly inhibited *ADAM17* expression in A549 cells. Lentivirus-mediated RNAi targeting of *ADAM17* significantly inhibited TNF-α production in the supernatants (*p*<0.05), whereas the level of TNF-α in the cells was increased (*p*<0.05). Lentiviral *ADAM17* RNAi inhibited MMP9 expression, IκBα phosphorylation and the expression of phosphorylation p65 protein in response to LPS (*p*<0.05). PDTC significantly inhibited the expression of MMP9 and the phosphorylation of IκBα, as well as the expression of phosphorylation p65 protein in response to TNF-α (*p*<0.05). Lentiviral RNAi targeting of *ADAM17* down-regulates LPS-induced MMP9 expression in lung epithelial cells via inhibition of TNF-α/NF-κB signaling.

## Introduction

Chronic obstructive pulmonary disease (COPD) is a leading cause of chronic morbidity and mortality that imposes a substantial economic and social burden worldwide. The permanent destruction of alveolar walls, which results in airspace enlargement, as well as the loss of elastic recoil and a decrease in surface area for gas exchange and lung hyperexpansion are hallmarks of COPD [1]. Protease–antiprotease imbalance is thought to play a key role in the pathogenesis of COPD. Matrix metalloproteinases (MMPs) are a family of proteolytic enzymes, which belong to the metazincin family and share the conserved zinc-binding motif in their catalytic active site [2]. MMPs not only actively participate in remodeling the extracellular matrix (ECM) by degrading certain constituents, but also are important regulators of extracellular tissue signaling networks, which may regulate cell proliferation, migration, differentiation, apoptosis and angiogenesis [2]–[4]. The ultimate impact of MMPs on ECM degradation may be regulated at several levels, including gene transcription, proenzyme activation and inhibition of active enzymes [5]. MMP9 also known as gelatinase B is involved in the degradation of elastin, aggrecan, and type IV, V and VII collagen [4]–[8], which may contribute to the development of COPD [9]. An increase in MMP9 has been detected respectively in the sputum and bronchoalveolar lavage fluid (BALF) of patients with COPD [10], [11]. Experimental animal models have also shown that over-expression of MMP9 induces pathological changes that are similar to those associated with emphysema, including airspace enlargement and loss of alveolar elastin in mice [6]. Moreover, higher serum concentrations of MMP9 have been linked to airway obstruction and COPD progression [12], [13]. However, the complex and highly orchestrated interactions between inflammatory cells, inflammatory cytokines and MMP9 are not well understood. Furthermore, the mechanism that regulates MMP9 expression in lung has not previously been clarified.

Tumor necrosis factor (TNF)-α is a potent pro-inflammatory cytokine that exerts pleiotropic effects on various cell types and plays a critical role in the pathogenesis of chronic inflammatory diseases, such as COPD [14]. In the classical nuclear factor-κB (NF-κB) signaling pathway, stimulation of TNF-α activates the inhibitor of NF-κB kinase (IKK) signalosome, which leads to the phosphorylation of inhibitor of NF-κB (IκB) on 2 conserved N-terminal serine residues. Phosphorylated IκB is then ubiquitinated and subsequently degraded by the S26 proteasome [15]. Finally, NF-κB is activated and translocated into the nucleus where it binds to a decameric consensus motif and facilitates the transcription of target genes. TNF-α is initially synthesized as a transmembrane protein (mTNF-α) with a molecular mass of 26 kDa that is cleaved to yield a 17 kDa soluble product (soluble TNF-α). A disintegrin and metalloproteinase 17 (ADAM17), also called TNF-α converting enzyme (TACE), plays a vital role during ectodomain shedding of TNF-α [16], [17]. Several studies have shown that NF-κB signal pathway mediates MMP9 expression that is induced by TNF-α [18], [19]. Therefore, we hypothesized that the ADAM17/TNF-α/NF-κB signaling pathway mediates MMP9 expression in lung epithelial cells.

RNA interference (RNAi) is a powerful research tool for studying gene function in vitro and in vivo [20]. Lentiviral vectors can efficiently transduce both dividing and nondividing cells to established sustained transgene expression. These properties make lentiviral vectors attractive vehicles for the delivery of small interfering RNA genes into mammalian cells [21]. Therefore, in the present study, we constructed lentiviral vectors targeting the human *ADAM17* gene, investigated whether TNF-α signaling mediated LPS-induced MMP9 expression, and explored the effects of lentiviral RNAi-mediated knockdown of *ADAM17* on TNF-α/NF-κB signaling and MMP9 expression in A549 lung epithelial cells treated with LPS.

## Materials and Methods

### Construction and production of lentiviral expression vectors

The human *ADAM17* mRNA sequence (GenBank accession number: NM_003183) was used to determine suitable siRNA target sequences and CCTATGTCGATGCTGAACAAA was selected. The siRNAs were converted into short hairpin RNA (shRNA) with a stem-loop-stem conformation and *ClaI* and *MluI* (Trono Laboratory, University of Geneva, Switzerland) restriction sites were added at the 5′ and 3′ ends, respectively. DNA oligonucleotides chemically synthesized by Sangon Biotech Co., Ltd. (Shanghai, China) were annealed and then inserted into pLVTHM vectors following double digestion with *ClaI* and *MluI* and ligation with T4 DNA ligase (NEB, Ipswich, MA, USA). The recombinant vectors were transformed into DH5α -competent Escherichia coli cells (Invitrogen, Carlsbad, CA, USA). The orientation of the inserted shRNA cassettes was verified by restriction enzyme analysis and DNA sequencing. A negative control (*NC*) siRNA sequence (TTCTCCGAACGTGTCACGT) was used as a control for *ADAM17* siRNA.

### Lentivirus production and transduction

293T cells (Purchased from Shanghai Institute of Biology, Chinese Academy of Sciences) were cultured in DMEM supplemented with 10% FBS. When cell fusion reached 80%, 20 µg recombinant pLVTHM vectors and packaging helper plasmids, including 10 µg pMD2G-VSVG, 10 µg pMDlg-pRRE and 10 µg pRsv-REV (Trono Laboratory, University of Geneva, Switzerland), were co-transfected into 293T cells with calcium phosphate. The medium was replaced with fresh culture medium 12 h after transfection. The cultured supernatants were collected and centrifuged at 800×g for 7 min at 4°C 48 h post-transfection to remove cell debris. The supernatants were then filtered through a 0.45 µm pore filter prior to ultra-centrifugation at 50000×g for 90 min at 4°C. Viral particles were precipitated in ice-cold PBS re-suspension solution. Finally, the viral particles, LV-*ADAM17*-shRNA and LV-*NC*-shRNA, were stored at −80°C. The viral titer was detected via infection of 293T cells and subsequent flow cytometric analysis. The titer of the recombinant virus was 2.16×108 TU/ml.

### Cell culture and treatment

A549 lung epithelial cells (Shanghai Institute of Biology, Chinese Academy of Sciences) were cultured at a concentration of 1×109 cells/l in Dulbecco's modified eagle medium (DMEM, GIBCO, Carlsbad, CA, USA) containing 10% FBS in a humidified atmosphere of 5% carbon dioxide and 95% air at 37°C. The cells were incubated for 30 min with 5 mg/l of a TNFR1 blocking peptide (TNFR1BP), chemically synthesized by Sangon Biotech Co., Ltd. (Shanghai, China), and with 50 mg/l pyrrolidine dithiocarbamate (PDTC; Sigma, St Louis, Missouri, USA). The cells were then stimulated with 100 µg/l LPS (Biosea, Beijing, China), and the supernatants and cells were collected. Conversely, A549 cells were infected with recombinant lentivirus at a multiplicity of infection of 15. At 72 h post-infection, the cells were stimulated for 24 h with LPS (100 µg/l) or TNF-α (Biosea, China) at a concentration of 1 mg/l. The cells and supernatants were then harvested.

### Reverse transcription polymerase chain reaction (RT-PCR)

Total RNA was extracted from the cells with RNAex reagent (Watson, Beijing, China) according to the manufacturer's instruction, and dissolved in nuclease-free water. The final RNA concentrations were determined with a spectrophotometer. First strand cDNA synthesis was performed with Moloney murine leukemia virus reverse transcriptase (M-MLV RT, Promega, USA). The PCR reactions were performed in a 25 µl reaction volume, which included 2.5 µl first-strand cDNA, 2 µl sense primer (10 µmol/l), 2 µl antisense primer (10 µmol/l), 2.5 µl 10×PCR buffer, 2 µl MgCl2 (25 mmol/l), 1 µl dNTP (10 mmol/l) and 0.5 µl Taq-DNA polymerase (Tiangen Biotech, China). The initial denaturation step was performed for 5 min at 95°C; 35 cycles followed, each consisting of denaturation for 1 min at 94°C, annealing for 1 min at 58.5°C, and extension for 1 min at 72°C. The final extension was performed for 5 min at 72°C. As an internal control, *ACTB* was amplified to normalize the starting amount of cDNA for each sample. The primers were designed as follows (AuGT Biotech, China): *ADAM17*(Genebank: NM_003183), 5′-GCTTGGCACACCTTTTCACATACC-3′(sense); reverse: 5′-GTCCTCATTCGGGGCACATTCTG-3′(antisense); *MMP9* (GenBank: NM-004994), 5′-TTGACAGCGACAAGAAGTGG-3′ (sense) and 5′-GGCACAGTAGTGGCCGTAG-3′ (antisense); *ACTB*, 5′-GGCACCACACCTTCTACAATGA-3′ (sense) and 5′-TCAGGAGGAGCAGCAATGATCTTG-3′ (antisense). The expected sizes of the PCR products were 287 bp for *ADAM17*, 388 bp for *MMP9* and 745 bp for *ACTB*. PCR products were visualized by electrophoresis on ethidium bromide stained 1.5% agarose gels, and the band densities were analyzed with a UVP-GDS8000 gel analysis system (Ultra-Violet Products Ltd., UK). The expression of *ADAM17* or *MMP9* mRNA were evaluated by determining the *ADAM17* or *MMP9* mRNA/*ACTB* mRNA ratio.

### Enzyme-linked immunosorbent assay (ELISA)

The amount of TNF-α in the supernatants was measured with ELISA kits (R&D Systems, Minneapolis, MN, USA), which were used in accordance with the manufacturer's instructions. Briefly, the supernatants were coated onto the 96-well plates and incubated overnight at 4°C. After blocking for 1 h at room temperature with blocking solution (1% FBS and 0.05% Tween-20 in PBS), monoclonal anti-human TNF-α was added to each well and incubated for 1 h at room temperature. After washing with 0.05% Tween-20 in PBS, peroxidase-conjugated immunopure goat anti-rabbit IgG (H+L) (Catalog# 31460; Pierce, Rockford, IL, USA) was added and incubated for 1 h at room temperature. A color reaction was developed with tetramethylbenzidine for 10 min at room temperature. Following the addition of a stop solution, absorbance at 450 nm was measured in a microtiter plate reader.

### Western blotting

Nuclear and cytoplasmic proteins were extracted from the cells with NE-PER Nuclear and Cytoplasmic Extraction Reagent (Catalog # 78833; Pierce, Rockford, IL, USA) according to the manufacturer's instructions. Protein concentration was measured with a protein assay kit (Micro BCA; Pierce, USA). Equal amounts of cell lysates containing 10 µg protein were incubated for 5 min in boiling water. Proteins were separated by 10% sodium dodecyl sulfate polyacrylamide gel electrophoresis and then electroblotted onto Hybond-ECL nitrocellulose membrane. The membrane was blocked with TBS solution containing 5% skim milk at room temperature for 1 h. Membranes were then incubated in primary antibodies for 1 h at room temperature on an orbital shaker. The membranes were then washed 5 times, for 5 min each, in Tris-buffered saline Tween (TBS-T) and incubated in diluted peroxidase-conjugated immunopure goat anti-rabbit IgG (H+L)(Catalog#31460; Pierce, USA) for 1 h at room temperature on an orbital shaker. After the membranes were again washed 5 times for 5 min each in TBS-T, the proteins were visualized with an enhanced chemiluminescence solution (Amersham Pharmacia biotech, Buckinghamshire, UK). The images were developed on X-ray film and the band densities were analyzed with a UVP-GDS8000 gel analysis system. The same membranes were stripped and blotted with anti-β-actin antibodies, which provided a loading control. The primary antibodies were obtained from the following sources: MMP9 (G657) Antibody (Catalog#2270), IκBα Antibody which can bind to IκBα that is not phosphorylated at Ser32/Ser36 (Catalog #9242), Phospho-IκBα (Ser32) (14D4) Rabbit mAb (Catalog#2859), TNF-α rabbit polyclonal antibody (Catalog#3707S) and phospho-NF-κB p65 (p-p65) (Ser536) Mouse mAb (Catalog# 3036S) from Cell Signaling Technology (Danvers, Massachusetts, USA); and β-actin (N-21) antibody (Catalog#sc130656) from Santa Cruz Biotechnology (Santa Cruz, California, USA). Protein markers (Catalog #7727) were obtained from Cell Signaling Technology (Danvers, USA).

### Statistical analyses

Data were analyzed for statistical significance via one-way analysis of variance (ANOVA), paired-sample *t*-tests, and multiple comparisons in ANOVA were analyzed via the Student-Newman-Keuls test. Data were expressed as the mean ± SD. Values of *p*<0.05 were considered statistically significant.

## Results

### LPS induced both dose- and time-dependent expression of MMP9 mRNA

**Figure 1:**
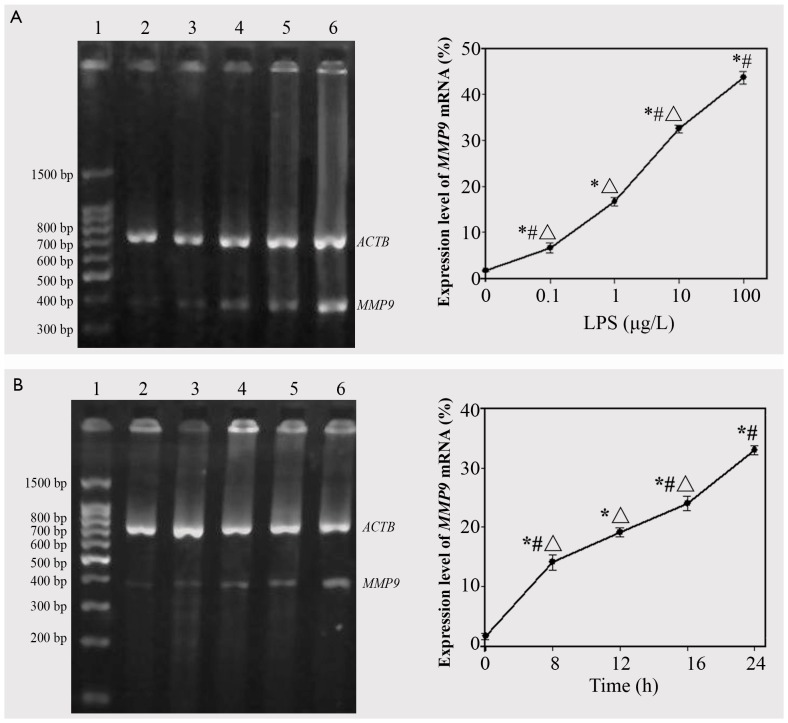
LPS-induced expression of *MMP9* mRNA in a dose- and time-dependent manner. (A) lane 1: DNA ladder marker; lane 2–6: *MMP9* mRNA expression in A549 cells stimulated with LPS at concentrations ranging from 0.1 to 100 µg/l. (B) DNA ladder marker; lane 2–6: expression of *MMP9* mRNA in A549 cells stimulated for different durations (0–24 h) with LPS at a concentration of 10 µg/l.

When A549 cells were stimulated for 24 h with LPS, at concentrations ranging from 0.1–100 µg/l, *MMP9* mRNA expression was induced by LPS in a dose-dependent manner (*p*<0.05; Fig. 1A). When A549 cells were stimulated for 0, 8, 12, 16, or 24 h with LPS, at a concentration of 10 µg/l, *MMP9* mRNA expression was induced by LPS in a time-dependent manner (*p*<0.05; Fig. 1B).

### LPS-induced MMP9 expression is mediated by the TNF-α/NF-κB signaling pathway in A549 cells

**Figure 2:**
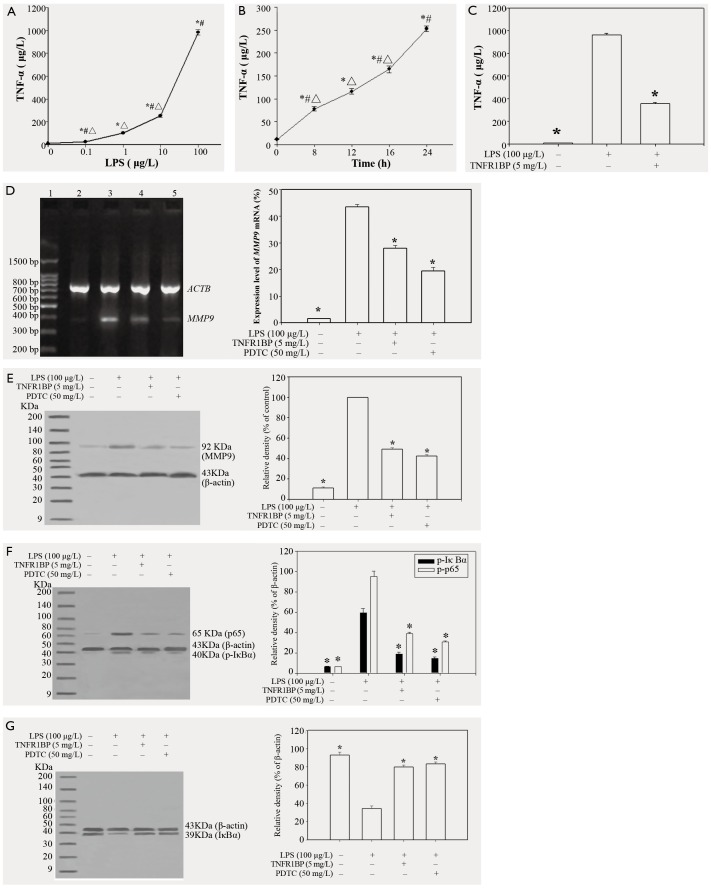
LPS-induced MMP9 expression is mediated by the TNF-α/NF-κB signaling pathway in A549 cells. (A) A549 cells were stimulated with LPS at concentrations ranging from 0.1 to 100 µg/l and TNF-α protein production in the supernatants was detected using ELISA. (B) A549 cells stimulated for different lengths of time (0–24 h) with LPS at a concentration of 10 µg/l. (C) A549 cells were pretreated for 30 min with 5 mg/l TNFR1BP and were then stimulated for 24 h with LPS at a concentration of 100 µg/l. TNF-α protein in the supernatants was detected via ELISA. (D) TNFR1BP and PDTC inhibited LPS-induced *MMP9* mRNA expression. Lane 1: DNA ladder marker; lane 2: the group not stimulated by LPS; lane 3: the group stimulated for 24 h with LPS at a concentration of 100 µg/l; lane 4: A549 cells were pretreated for 30 min with 5 mg/l TNFR1BP and then stimulated for 24 h with LPS at a concentration of 100 µg/l; Lane 5: A549 cells were pretreated for 30 min with 50 mg/l PDTC and then stimulated for 24 h with LPS at a concentration of 100 µg/l. (E) TNFR1BP and PDTC inhibited LPS-induced MMP9 expression. A549 cells were pretreated for 30 min with 5 mg/l TNFR1BP and 50 mg/l PDTC and then stimulated for 24 h with LPS at a concentration of 100 µg/l. The expression of MMP9 protein was detected via western blotting. (F) The effects of TNFR1BP and PDTC on LPS-induced the phosphorylation of IκBα and p65. A549 cells were pretreated for 30 min with 5 mg/l TNFR1BP and 50 mg/l PDTC and then stimulated for 24 h with LPS at a concentration of 100 µg/l. Both the phosphorylation of IκBα and p65 was detected via western blotting. (G) The effects of TNFR1BP and PDTC on IκBα protein expression in the cytoplasm. A549 cells were pretreated for 30 min with 5 mg/l TNFR1BP and 50 mg/l PDTC and then stimulated for 24 h with LPS at a concentration of 100 µg/l. The level of IκBα in the cytoplasm was detected via western blotting.

The expression of TNF-α protein in the supernatants was induced by LPS in a dose- and time-dependent manner in A549 cells (*p*<0.05; Fig. 2A and 2B). When the cells were pretreated for 30 min with TNFR1BP, and then stimulated with LPS for 24 h, the expression of TNF-α protein was significantly decreased (*p*<0.05; Fig. 2C). The phosphorylation of IκBα and p65 protein expression was induced by stimulation with LPS. But the level of IκBα (unphosphorylated) in the cytoplasm was significantly decreased under the stimulation of LPS (*p*<0.05; Fig. 2G). When the cells were pretreated for 30 min with TNFR1BP and PDTC, and then stimulated for 24 h by LPS, the level of the IκBα in the cytoplasm was significantly increased (*p*<0.05; Fig. 2G), and both the phosphorylation of IκBα and expression of p-p65 protein were significantly inhibited (*p*<0.05; Fig. 2F). LPS-induced MMP9 expression was also markedly inhibited by TNFR1BP and PDTC (*p*<0.05; Fig. 2D and 2E).

### Effects of lentivirus-mediated ADAM17 RNAi on the expression of MMP9 and p-p65 protein, and the phosphorylation of IκBα, in response to LPS

**Figure 3:**
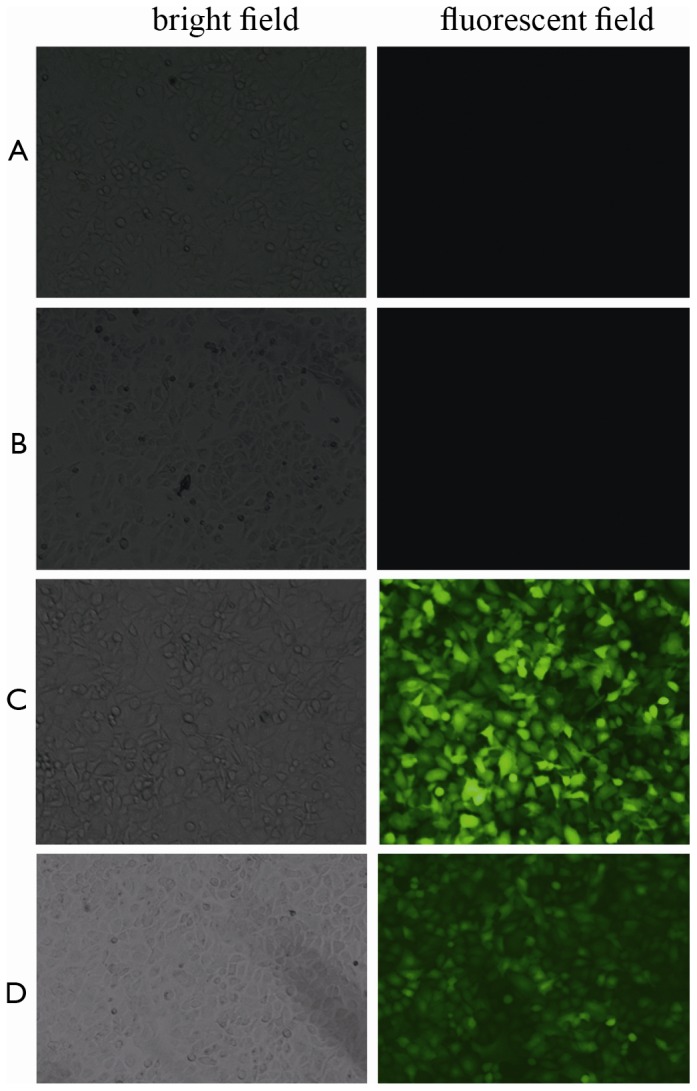
Microfluorographs of A549 cells 96 h after transfection. A: A549 cells not stimulated with LPS. B: A549 cells only stimulated for 24 h with LPS at a concentration of 100 µg/l. C: A549 cells transfected for 72 h with LV-*NC*-shRNA and then stimulated for 24 h with LPS at a concentration of 100 µg/l. D: A549 cells were transfected for 72 h with LV-*ADAM17*-shRNA and then stimulated for 24 h with LPS at a concentration of 100 µg/l.

**Figure 4:**
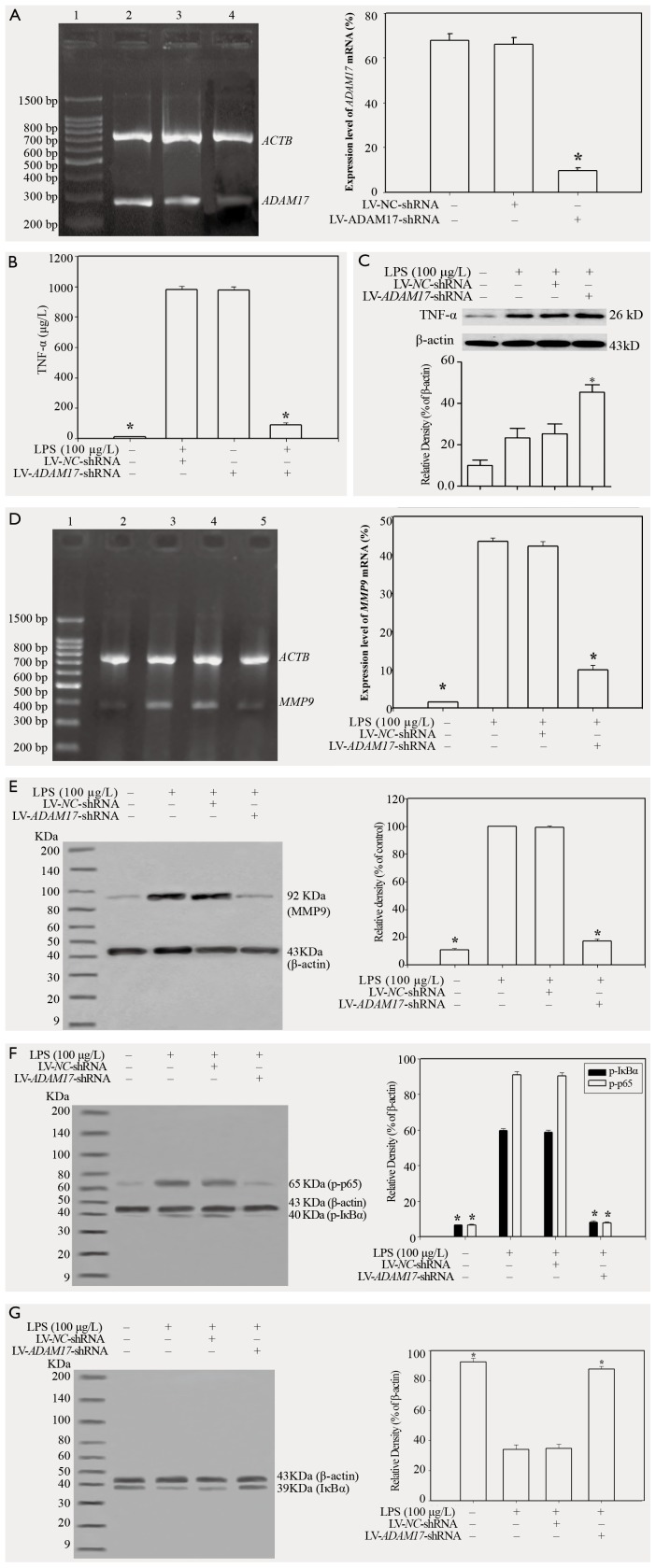
Effects of lentivirus-mediated ADAM17 RNAi on the expression of MMP9 and p-p65 protein, and the phosphorylation of IκBα, in response to LPS. (A)The silencing effects of lentivirus mediated RNA interference on ADAM17 expression in A549 cells. (B) The effects of lentiviral ADAM17 RNA interference on LPS-induced TNF-α protein production in the supernatants. A549 cells were infected for 72 h with LV-ADAM17-shRNA or LV-NC-shRNA and then stimulated for 24 h with LPS (100 µg/l). (C) The effects of lentiviral ADAM17 RNA interference on TNF-α shedding in A549 cells. The level of TNF-α in the A549 cells was detected by western blotting. (D) The effects of lentiviral ADAM17 RNA interference on LPS-induced expression of MMP9 mRNA. Lane 1: DNA ladder marker; lane 2: the group not stimulated with LPS; lane 3: A549 cells stimulated for 24 h with LPS (100 µg/l); lane 4: A549 cells were infected for 72 h with LV-NC-shRNA and then stimulated for 24 h with LPS (100 µg/l); lane 5: A549 cells were infected for 72 h with LV-ADAM17-shRNA and then stimulated for 24 h with LPS (100 µg/l). (E) The effects of lentiviral ADAM17 RNA interference on LPS-induced expression of MMP9 protein. A549 cells were infected for 72 h with LV-ADAM17-shRNA or LV-NC-shRNA and then stimulated for 24 h with LPS at a concentration of 100 µg/l. (F) The effects of lentiviral ADAM17 RNA interference on LPS-induced phosphorylation of IκBα and p65. A549 cells were infected for 72 h with LV-ADAM17-shRNA or LV-NC-shRNA and then stimulated for 24 h with LPS at a concentration of 100 µg/l. Both the phosphorylation of IκBα and p65 was detected via western blotting. (G) The effects of lentiviral ADAM17 RNA interference on IκBα protein expression in the cytoplasm. A549 cells were infected for 72 h with LV-ADAM17-shRNA or LV-NC-shRNA and then stimulated for 24 h with LPS at a concentration of 100 µg/l. The level of IκBα in the cytoplasm was detected via western blotting.

A549 cells were infected with LV-*ADAM17*-shRNA or LV-*NC*-shRNA for 72 h (Fig. 3), and lentivirus-mediated *ADAM17* RNAi significantly inhibited *ADAM17* mRNA expression in A549 cells (*p*<0.05; Fig. 4A). When A549 cells were infected with LV-*ADAM17*-shRNA or LV-*NC*-shRNA for 72 h, and then stimulated for 24 h with LPS at 100 µg/l, the production of TNF-α protein in the supernatants was significantly decreased by LV-*ADAM17*-shRNA (*p*<0.05; Fig. 4B), on the contrary the levels of TNF-α protein in A549 cells were significantly increased (*p*<0.05; Fig. 4C). MMP9, phosphorylated IκBα, as well as p-p65 were markedly inhibited by LV-*ADAM17*-shRNA (*p*<0.05; Fig. 4D, 4E, 4F). When the cells were infected with LV-*ADAM17*-shRNA for 72 h, and then stimulated for 24 h with LPS, the level of IκBα (unphosphorylated) in the cytoplasm was significantly increased (*p*<0.05; Fig. 4G). The results also indicated that LV-*NC*-shRNA had no effect on the production of TNF-α in response to LPS (*p*>0.05; Fig. 4B). LPS-induced MMP9 expression, IκBα phosphorylation and p-p65 expression could not be inhibited by LV-*NC*-shRNA in A549 cells (*p*>0.05; Fig. 4D, 4E, 4F).

### Effects of lentiviral ADAM17 RNAi on MMP9 expression, IκBα phosphorylation, and p-p65 protein expression induced by TNF-α

**Figure 5:**
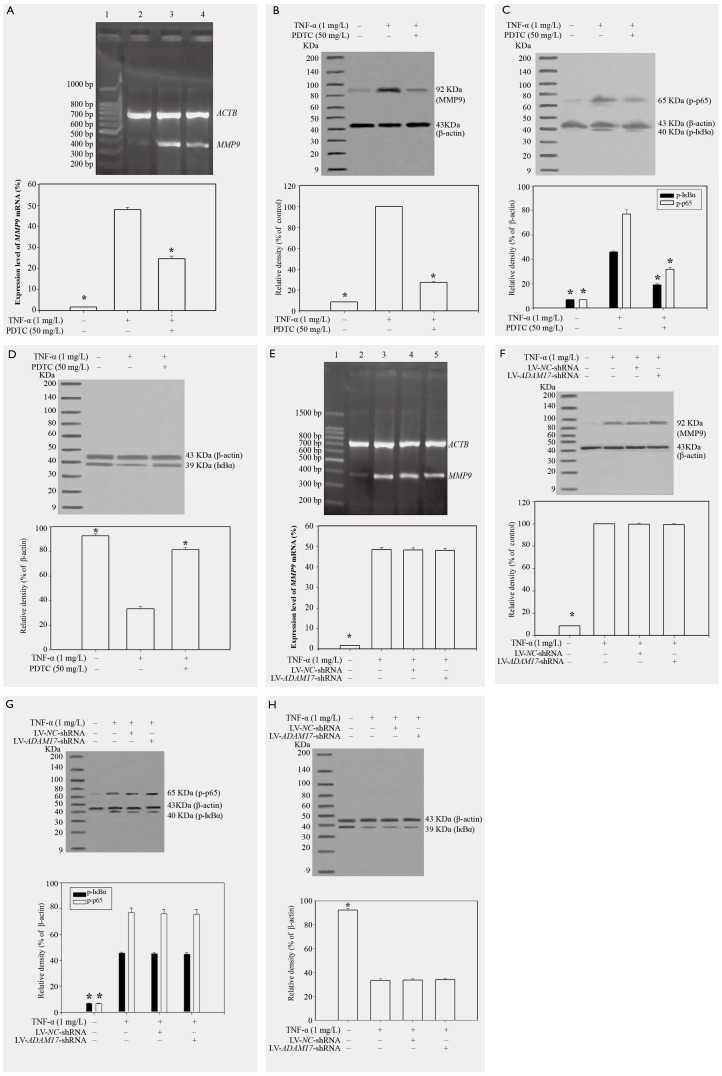
Effects of lentiviral ADAM17 RNAi on MMP9 expression, IκBα phosphorylation, and p-p65 protein expression induced by TNF-α. (A) PDTC inhibited TNF-α-induced expression of MMP9 mRNA in A549 cells. Lane 1: DNA ladder marker; lane 2: cells not stimulated with TNF-α; lane 3: cells stimulated for 24 h with TNF-α at a concentration of 1 mg/l; lane 4: cells pretreated for 30 min with 50 mg/l PDTC and then stimulated for 24 h with 1 mg/l TNF-α. (B) The effects of PDTC on TNF-α-induced expression of MMP9 protein. A549 cells were pretreated for 30 min with 50 mg/l PDTC and then stimulated for 24 h with 1 mg/l TNF-α. (C) The effects of PDTC on the phosphorylation of IκBα and p65 induced by TNF-α. A549 cells were pretreated for 30 min with 50 mg/l PDTC, and then stimulated for 24 h by TNF-α at a concentration of 1 mg/l. (D) The effects of PDTC on IκBα protein expression in the cytoplasm. A549 cells were pretreated for 30 min with 50 mg/l PDTC and then stimulated for 24 h with 1 mg/l TNF-α. The level of IκBα in the cytoplasm was detected via western blotting. (E) The effects of lentiviral ADAM17 RNA interference on TNF-α-induced expression of MMP9 mRNA in A549 cells. Lane 1: DNA ladder marker; lane 2: cells not stimulated with TNF-α; lane 3: cells stimulated only with 1 mg/l TNF-α; lane 4: the cells were infected for 72 h with LV-NC-shRNA and then stimulated for 24 h with 1 mg/l TNF-α; lane 5: cells infected for 72 h with LV-ADAM17-shRNA and stimulated for 24 h with 1 mg/l TNF-α. (F) The effects of lentiviral ADAM17 RNA interference on TNF-α-induced expression of MMP9 protein. A549 cells were infected for 72 h with LV-ADAM17-shRNA or LV-NC-shRNA and then stimulated for 24 h with 1 mg/l TNF-α. (G) The effects of lentiviral ADAM17 RNA interference on induced by TNF-α-induced phosphorylation of IκBα and p65. A549 cells were infected for 72 h with LV-ADAM17-shRNA or LV-NC-shRNA and then stimulated for 24 h with TNF-α (1 mg/l). (H) The effects of lentiviral ADAM17 RNA interference on IκBα protein expression in the cytoplasm. A549 cells were infected for 72 h with LV-ADAM17-shRNA or LV-NC-shRNA and then stimulated for 24 h with 1 mg/l TNF-α. The level of IκBα in the cytoplasm was detected via western blotting.

When A549 cells were pretreated for 30 min with 50 mg/l PDTC, and then stimulated for 24 h with TNF-α at 1 mg/l, PDTC markedly inhibited the phosphorylation of IκBα and p65 in response to TNF-α in A549 cells (*p*<0.05; Fig. 5C), and the level of IκBα (unphosphorylated) in the cytoplasm was significantly increased (*p*<0.05; Fig. 5D) Correspondingly, TNF-α-induced MMP9 expression was markedly inhibited by PDTC (*p*<0.05; Fig. 5A, 5B). When A549 cells were infected with recombinant lentivirus for 72 h, and then stimulated for 24 h with TNF-α at 1 mg/l, LV-*ADAM17*-shRNA and LV-*NC*-shRNA had no effect on the expression of MMP9, the level of unphosphorylated IκBα and the phosphorylation of IκBα and p65 in response to TNF-α (*p*>0.05; Fig. 5E, 5F, 5G, 5H).

## Discussion

MMP9 is not produced by resident cells in the normal lung; however, in response to various forms of stimulation, MMP9 is expressed, produced, and secreted by many inflammatory cells and some airway structural cells [22], [23]. LPS, or endotoxin, is a predominant, integral structural component of the outer membrane of gram-negative bacteria and one of the most potent microbial initiators of inflammation [24]. In the present study, the expression levels of *MMP9* mRNA and MMP9 protein were significantly increased by the stimulation of LPS in A549 lung epithelial cells. The transcription factor NF-κB is a dimeric complex composed of members of the Rel protein family, which includes p65 (RelA), p105/p50, p100/p52, RelB, c-Rel, and the viral oncoprotein (v-Rel). In its classic form, NF-κB is a dimer that consists of the transcriptionally inactive p50 subunit and the p65/RelA (p65) subunit. IκB is responsible for regulating the DNA-binding activity and nucleo/cytoplasmic distribution of NF-κB [25]. The most well characterized IκB protein is IκBα [25], [26]. Association of IκBα with NF-κB disrupts DNA binding and masks the nuclear localization signals (NLSs) located in the C-terminal region of the Rel homology domain (RHD). Masking the NLSs is believed to impede the nuclear translocation of NF-κB, which results in the retention of NF-κB in the cytoplasm where it cannot mediate any transcriptional effects. Any of several extracellular stimuli can activate NF-κB by initiating a signal transduction pathway that leads to phosphorylation, ubiquitination and, ultimately, degradation of IκBα [25]. Once IκBα is degraded, the NLSs within the RHD are unmasked, allowing NF-κB to enter the nucleus and initiate transcription of target genes [27]. PDTC, which can protect IκBα from degradation, is a special inhibitor of NF-κB [28]. TNFR1BP can bind to TNF-α and block the biological activities mediated by TNF-α [29]. In the present study, the IκBα antibody (binding to IκBα that is not phosphorylated at Ser32/Ser36) was used to detect the levels of IκBα protein in the cytoplasm. The results showed that the level of IκBα which is not phosphorylated at Ser32/Ser36 in the cytoplasm was significantly decreased under the stimulation of LPS. When the cells were pretreated for 30 min with TNFR1BP and PDTC, and then stimulated for 24 h by LPS, the level of IκBα in the cytoplasm was significantly increased. In the present study, TNFR1BP inhibited the expression of TNF-α and the phosphorylation of IκBα and p65 in response to LPS. Furthermore, LPS-induced the phosphorylation of IκBα and p65 was also inhibited by PDTC. Accordingly, LPS-induced MMP9 expression was markedly decreased when the cells were pretreated with TNFR1BP and PDTC. Therefore, the TNF-α/NF-κB signaling pathway mediated LPS-induced MMP9 expression in A549 lung epithelial cells.

The ADAMs are a family of transmembrane and secreted proteins with a variety of functions, including proteolytic cleavage of cell surface molecules, cell fusion, cell adhesion and intracellular signaling [30]. ADAM17, a member of ADAM family, has been described as “a signaling scissor”. The most well-known function of catalytically active ADAM17 is to cleave the ectodomains of various transmembrane proteins, such as growth factors, receptors and their ligands, cytokines, and cell adhesion molecules; therefore, ADAM17 is an important regulator of almost every cellular event [31], [32]. Though MMP7, MMP14, MMP17 and ADAM10 are also involved in the ectodomain shedding of TNF-α, the specificity constants for cleavage of TNF-α are approximately 100–1000-fold lower than ADAM17 [17]. Therefore, ADAM17 is a key sheddase in the proteolytic cleavage of the extracellular domain of mTNF-α [33]. RNAi has received attention for being one of the most powerful tools for reverse genetics in the post-genome era. Several groups have developed vector-based siRNA expression systems that can induce RNAi in living cells. With lentiviral vectors for shRNA expression, high transduction efficiencies can be achieved, which minimizes the effects of clonal selection of phenotypically diverse cells [34]. Therefore, *ADAM17* shRNA expression vectors were constructed in the present study, and *ADAM17* expression was significantly inhibited by lentivirus-mediated *ADAM17* RNAi in A549 cells. Lentiviral *ADAM17* RNAi inhibited the expression of TNF-α protein in the supernatants, whereas the level of TNF-α in the cells were increased, which indicated that ADAM17 mediated the shedding of TNF-α. In the present study, lentiviral *ADAM17* RNAi inhibited the phosphorylation of IκBα and p65 in response to LPS, and the level of IκBα which is not phosphorylated at Ser32/Ser36 in the cytoplasm was significantly increased. Accordingly, LPS-induced MMP-9 expression was markedly decreased. In contrast, lentiviral *ADAM17* RNAi had no effect on the TNF-α-induced phosphorylation of IκBα and p65. Furthermore, *ADAM17* RNAi did not inhibit TNF-α-induced MMP9 expression in A549 lung epithelial cells. These results indicated that ADAM17 RNAi does not directly inhibit downstream TNF-α signaling, and that ADAM17 mediates LPS-induced MMP9 expression in lung epithelial cells by regulating soluble TNF-α.

In conclusion, TNF-α/NF-κB signaling plays an important role in LPS-induced MMP9 expression in lung epithelial cells. Furthermore, lentiviral RNAi targeting of *ADAM17* down-regulated LPS-induced MMP9 expression via inhibition of TNF-α/NF-κB signaling, which provides a potential target for therapeutic intervention in COPD.
